# Intraoperative Ethanol Treatment as an Adjuvant Therapy of Pterygium Excision

**Published:** 2006-12

**Authors:** Ko-Hua Chen, Wen-Ming Hsu

**Affiliations:** 1 *Department of Ophthalmology, Taipei Veterans General Hospital, Taipei, Taiwan (ROC);*; 2 *National Yang-Ming University 201, Section II, Shih-Pai Road, Taipei, Taiwan (ROC);*; 3 *Division of Medical Engineering, National Health Research Institutes. Taipei, Taiwan (ROC)*

**Keywords:** ethanol, excision, pterygium, eecurrence, mitomycin

## Abstract

Recurrence of pterygium is the main concern for ophthalmic surgeons after the excision of pterygium. To evaluate the efficacy and safety of ethanol treatment during pterygium excision in preventing the recurrence of pterygia. A prospective randomized study was performed of 78 eyes in primary pterygium patients treated by excision. Primary pterygium patients were randomly assigned to ethanol group (38 eyes given intraoperative ethanol) or mitomycin-c (MMC) group (40 eyes given intraoperative MMC). Ethanol (20%) was applied for 60 seconds to the pterygial and its adjacent corneal surfaces before pterygium excision. After excision, the excised site of sclera was soaked with 20% Ethanol for 60 seconds. In group 2, MMC (0.25 mg/ml) was applied for 60 seconds to the bare sclera after pterygium excision. The outcomes were followed for more than one year. Pterygium recurred in 2 (5.3%) of 38 eyes in ethanol group and 4 (10.0%) of 40 eyes in MMC group. Final appearance of the pterygium excision area was satisfactory in 73.6% of group1 and 67.5% of group 4. No patients experienced severe complications postoperatively. In comparison with MMC treatment, intraoperative ethanol is more efficacious in preventing recurrence of pterygium and causes fewer complications. It suggests this regimen as an alternative for the treatment of pterygium, especially for those patients of high risk group for MMC treatment complications.

## INTRODUCTION

Pterygia are benign fibrovascular and infiltrative processes of the corneal-conjunctival junctions characterized by a fleshy outgrowth of altered limbal/conjunctival tissue over the cornea. Pterygium can threaten human vision by central corneal invasion. Its high recurrence rate after initial excision is the major concern for ophthalmic surgeons and has lead to numerous methods to alleviate this problem. The bare sclera procedure described by Ombrain (1) has been practiced worldwide as the most basic technique for pterygium removal. However, the recurrence rate after this procedure is unacceptably high, 30–89% (2-4). In search of a more effective and safe method for preventing pterygium recurrence after surgical excision, many adjuvant therapies have been investigated such as Sr-90 beta-radiation (6), soft X-ray irradiation (7) intraoperative and post-operative mitomycin C (MMC) (3, 4, 8-10), subconjunctival injection of steroid (11) or 5-fluorouracil (12), conjunctival or limbal-conjunctival autograft transplantation, (13, 14), amniotic membrane transplantation (15, 16), split thickness buccal mucous membrane grafts (17), and excimer laser treatment (18). Many possible models of pterygium formation have been proposed, in which immunological reactions (20-23), potential oncogenic virus infections (24-26), degeneration processes (26, 27), ultraviolet irradiation, and neoplastic mechanisms (19, 25, 28, 29) participate in a multi-step process of pterygium pathogenesis. The variation in the theories of pterygium formation implies much about the pathogenesis of pterygium remains to be investigated.

To minimize the high recurrence rate after pterygium excision, many medications that inhibit cell proliferation (36, 37) have been applied to the excised sites. Intra-operative and post-operative MMC, an antibiotic antineoplastic agent that selectively inhibits the synthesis of DNA, RNA, and protein, were investigated and shown to be useful (4, 8-10). Intra-operative MMC has gained increasing acceptance in preventing pterygium recurrence (38) but unfortunately, MMC, a potent inhibitor of cell proliferation with cytotoxicity, caused complications especially in patients of high risk group such as ocular surface disorder and tear dysfunction. Alternative regimens remained to be identified (38).

Ethanol has been used widely in medical practice for its anti-microbial properties and also used in treating some tumors such as liver cancers because it causes massive cell apoptosis (39). Furthermore, it is known to cause in tissues the rapid denaturation of proteins/peptides including cytokines, enzymes, and growth factors that are supposed to be involved in pterygium formation and recurrence after excision. The application of ethanol to the cornea has been done in excimer laser refractory surgeries for around a decade. Low-dose (10–30%) ethanol treatment of the corneal epithelium for 20 to 30 seconds during the laser procedures was performed to remove corneal epithelium in photorefractive keratectomy and create corneal epithelial flaps in laser epithelial keratomileusis (LASEK) (40). It was shown that the application of ethanol at a concentration lower than 20% on the cornea for less than one minute appears to be safe (53-55). Most corneal epithelial cells were found to be alive by vital staining after low-dose ethanol treatment and the postoperative wound healing was normal (41). These studies, therefore, prompted us to conduct a prospective clinical trial to assess the safety and efficacy of intraoperative ethanol (20%) treatment, before and also after pterygium excision, for preventing recurrence. The efficacy of low-dose intraoperative ethanol was compared with that of intraoperative MMC in a historical control group of primary pterygium patients.

## PATIENTS AND METHODS

The protocol of this study was approved by the Institutional Review Board of Taipei Veterans General Hospital and the consent of these patients was obtained before the operations.

### Patient Population

In the prospective arm of this study, we performed bare sclera pterygium resection in a total of 78 eyes (78 patients) treated consecutively for primary pterygium at the Taipei Veterans General Hospital (Taipei, Taiwan, ROC) between April 2000 and October 2004. Those with small pterygia, <1.5 mm on the cornea, and those with meibomitis, blepharitis, acne rosacea, atopic keratoconjunctivitis, keratoconjunctivitis sicca, Sjögren’s syndrome, or herpes keratitis, or a history of previous ocular surgery, long-term application of ocular medications, or contact lens wear were excluded. Each patient underwent a complete ocular examination, including slit-lamp biomicroscopy and tear film examination.

**Figure 1:**
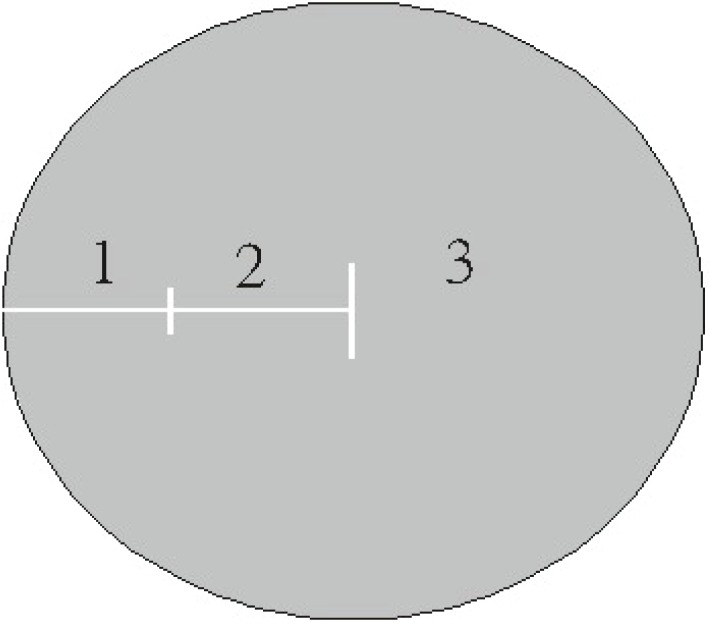
Classification system for pterygia grading. Circle represents cornea; 1, 2, and 3 represent grades of pterygia as they encroach onto cornea beyond the limbus.

To compare the efficacy of intraoperative ethanol and MMC, 38 eyes (38 patients) with primary pterygium (ethanol group) were enrolled randomly for treatment with ethanol and 40 eyes (40 patients) (MMC group) for treatment with MMC. A complete eye examination was performed by one investigator (WM Hsu) before surgery and 1 day, 3 days, 1 week, 10 days, 2 weeks, 1 month, 2 months, 3 months, 6 months and 1 year after surgery. To be included in this study, patients needed to return for all follow-up examinations. Severity of the pterygia was graded according to the extent of corneal encroachment beyond the limbus, as described by Wong V., *et al*. (43) (Figure 1). The distribution of pterygia by grade is given in Table 1. Characteristics of patients in each group are shown in Table 2.

**Table 1:** Number (percentage) of Pterygia Graded by Severity in two Groups

	Grade 1	Grade 2	Grade 3	Total

Group 1	18 (47.3%)	15 (39.5%)	5 (13.2%)	38
Group 2	16 (44.4%)	13 (36.1%)	7 (19.4%)	36

**Table 2:** Characteristics of pterigium treatment groups

Characteristics	Group 1	Group 2	*p*

Pterygium	Primary	Primary	
Adjuvant Therapy	Ethanol	MMC	
Number of eyes	38	40	
Number of patients	38	40	
Age^a^ (y)	60.8 ± 7.8 (45-84)	63.1 ± 9.0 (48-79)	0.18^b^
Gender (% Male)	53.3	46.6	0.96^c^

a Mean ± SD (range);

b Kruakal-Wallis test;

c Chi-square test.

Groups are described in the patients and Methods section. Ethanol, 20% intraoperatively for 60 seconds; MMC, 0.04% mitomycin C intra-operatively for 60 seconds.

Recurrence of pterygium is usually an early complication after the excision (44, 45) and was almost exclusively noted between post-operative 2-6 months in previous studies (44, 45). In a study by Hirst *et al*. (46), nearly 50% of recurrences occurred within the first 4 months and nearly all occurred within 1 year of pterygium removal. So all the subjects in this study were followed post-operatively at least for 12 months and we chose a 1-year cutoff period (47).

### Complete Corneal Epithelial Healing, Grading of Pterygium, the Final Appearance, and Definition of Pterygium Recurrence

The time needed for complete corneal epithelial healing was recorded and the final appearance of each patient’s operated eye was graded by the same investigator (WM Hsu), who was unaware of the group assignment. Corneal epithelium was checked by slit lamp microscope at every follow-up visit, and negative fluorescein staining of the cornea was defined as complete corneal epithelial healing.

Final appearance was checked by review of the last available photograph of the eyes using the criteria described by Prabhasawat *et al*. (48) In brief, a grade 1 result denoted the indistinguishability of the operated eye from normal; grade 2 was indicated by the presence of some fine episcleral vessels in the excised area extending up to the limbus but not beyond, in the absence of any fibrous tissue; grade 3, by the presence of additional fibrous tissues in the excised area without invasion into the cornea; and grade 4, by the true recurrence of pterygium with fibrovascular tissue invading a clear cornea. Pterygium recurrence rates, grade 4, are based on the results of the latest follow-up examination. Patients with grades 1 or 2 as their final results of pterygium excision were generally pleased with the postoperative appearance of their eyes.

### Surgical Techniques

**Figure 2:**
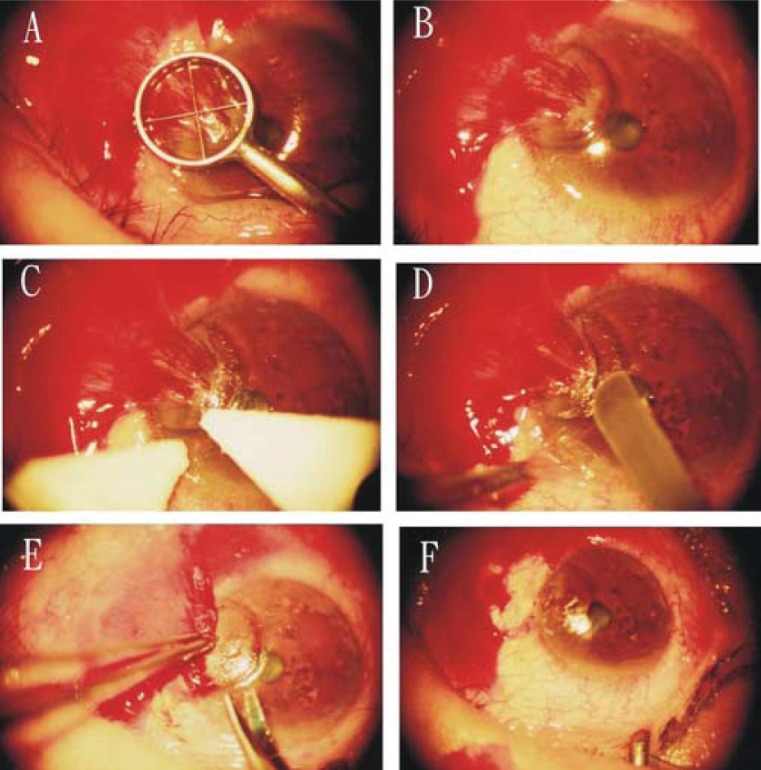
Technique for pterygium excision with intraoperative application of ethanol as an adjuvant. (A). Application of 20% ethanol in a ring marker. (B). Removal of the marker after absorbing the ethanol with a cellulose sponge. (C). Lifting the margin of epithelial flap with two cellulose sponges. (D). Dissecting the adherence of the pterygium body and separating with a Beaver 64 surgical blade. (E). Circumcising completely the conjunctival part of pterygium using a Westcott scissors along the mark. (F) A bare sclera after complete removal of residual pterygium tissue.

**Figure 3:**
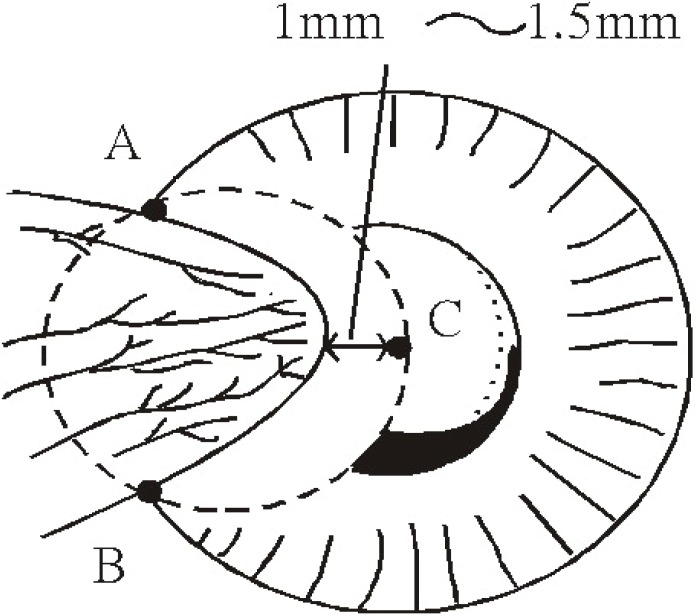
Determination of the adequate size of the optical zone marker. A optical zone marker (model E9011 3.0:storz. St. Louis, MO) of adequate size was used to delineate the area for corneal epithelium/pterygium removal. The size of the optical zone marker was determined by three points: point A and B, the pterium margins at limbus, and a third point, C, located 1 mm (in the case of primary pterygium) or 2 mm (in the case of recurrent pterygium) central to the head of pterygium.

All surgical procedures were performed by one surgeon (K. H. Chen) to ensure consistency in surgical methods. The procedure of ethanol application was modified from the LASEK method originally described by Massino Camellin, MD (M. Cimberele, “LASEK Has More Than 1 Year of Successful Experience”, Ocular Surgery News, July 15, 2000, pp. 14-17). The methods of MMC treatment were the same as those described by Wong VA *et al*. (43) Preparations for the surgery and the technique for pterygium excision were the same in all cases. All eyes were treated as follows (Figure 2):

- The ocular surface was anesthetized with 4 to 5 drops of topical 2% lidocaine (Xylocaine; Fujisawa Pharmaceutical, Osaka, Japan) in the preoperative holding area.
- After the eye was prepared with povidone-iodine (Betadine) and a plastic drape and lid speculum were placed, the pterygium was insufflated with a 2% lidocaine/1:100,000 epinephrine mixture to separate it from the underlying sclera.
- A Westcott scissors was used to separate the body of the pterygium (the scleral part of pterygium) from the surrounding conjunctiva, leaving the corneal part of pterygium attached on three sides.
- In the ethanol treatment group, an optical zone marker (model E9011 3.0:storz. St. Louis, MO) of adequate size was used to delineate the area for corneal epithelium/pterygium removal (Figure 2A). The size of the optical zone marker was determined by three points: point A and B, the pterygium margins at limbus, and a third point, C, located 1 to 1.5 mm (Figure 3).
- The barrel of the optical zone marker was filled with two drops of 20% ethanol. The dehydrated alcohol (1-ml ampoules; American Reagent Laboratories, Inc. Shirley. NY) was diluted in balanced salt solution and an ampoule opened fresh every treatment session. After 60 seconds, the ethanol was absorbed using a cellulose sponge (Medtronic Xomed, Jacksonville, FL) (Figure 2B).
- The loosened epithelium was lifted and removed, usually as a single sheet, with another dry cellulose sponge toward the pterygium site (Figure 2C).
- The adherence of the pterygium body was dissected and separated with a Beaver 64 surgical blade (Becton Dickinson, Franklin Lakes, NJ) and usually the epithelium/pterygium complex could be totally detached from the cornea together when the dissection reached the limbus.
- A Westcott scissors was used for complete circumcision of the conjunctiva along the mark (Figure 2E). Blunt dissection was used to free and resect the conjunctiva and Tenon’s capsule and fibrous tissue completely, leaving the bare sclera (Figure 2F) exposed. Complete removal of residual pterygium tissue was confirmed by the exposure of all episcleral blood vessels. Residual abnormal or scarred tissue on the corneal surface was scraped off with the same Beaver 64 surgical blade.
- In Ethanol group, at the completion of pterygium excision, 9 pieces of 5 × 5 mm Merocel sponge (Merocel Corp., Mystic, CT) supersaturated with 20% ethanol was applied to the bare scleral bed for 60 seconds followed by light irrigation with a 15-ml bottle of balanced salt solution (BSS).
- In MMC group, without any application of intraoperative ethanol, at the completion of pterygium excision, a 7 × 7 mm Merocel sponge (Merocel Corp., Mystic, CT) supersaturated with MMC (0.25 mg/mL; Kyowa Hakko Kogyo Co., Tokyo, Japan) was applied to the bare scleral bed for 60 seconds followed by light irrigation with BSS.
- No sutures were placed to approximate the excised edges of the conjunctiva in any patient.
- At the completion of the surgery in all groups, one drop of 0.3% gentamicin was applied topically to each operated eye and the eye was patched for 1 day.

Postoperatively, all eyes were treated with topical dexamethasone (Maxidex; Alcon, Puurs, Belgium) alone four times a day for 1 week, followed by tapered dosages until cessation of treatment at the end of the fourth week after surgery.

### Statistical Analysis

Student’s t-tests, Mann-Whitney U tests, Kruskal-Wallis tests and chi-square tests were used to compare groups. Fisher’s exact test was used to compare epithelial healing time, complication and recurrence rates between groups. The null hypothesis was rejected if the p value was <0.05. A StatView statistical software package (Abacus Concepts, Berkeley, CA) was used to analyze the data.

## RESULTS

All the patients in this study were Chinese and from Taiwan. The distribution of pterygia by grade is given in Table 1, and no statistical difference in grade was found between ethanol group and MMC treatment group (*p*>0.1). As Table 2 shows, there were no statistically significant differences in age or gender distribution among these four groups (*p*=0.18 and 0.96, respectively). The mean follow-up period for all groups in this study was 14.8 ± 3.8 months. (range, 12-20 months)

### Recurrence Rate

The recurrence rates of pterygium after excision were higher in the group treated with MMC than ethanol. Pterygium recurred within 1-12 months after surgery in 1 (2.6%) of 38 eyes of ethanol group and in 4 (10.0%) of 40 eyes of MMC group. But, using Fisher’s exact test, there was no significant difference (*p*=0.16) between patients in the ethanol treatment group and in the MMC treatment group.

The time to detection of pterygium recurrence after surgery in this study ranged between 2 and 12 months (3.7 ± 3.6 months) overall and was 3.0, 4.1 ± 1.7, 3.9 ± 2.1, and 4.5 ± 3.5 months for groups 1 and 2. There was also no difference between the two groups (*p*=0.32).

### Complete Corneal Epithelial Healing

The durations for complete corneal epithelial healing after surgeries are shown in Table 3. The time needed for epithelial healing was shorter in ethanol group (*p*<0.05), compared to that needed for healing of patients treated with MMC.

**Table 3:** Final outcome of 1ntraoperative ethanol and MMC for primary pterygia

	Group 1	Group 2	*P*

Follow up (Mo)^a^	15.3 ± 2.3 (12--18)	13.5 ± 3.3 (12--19)	>0.05
Time for complete corneal epithelial heal (days)^a^	3.3 ± 0.2 (3-5)	7.1 ± 1.7 (6-11)	<0.05
Grading of final appearance			
1	13/38 (34.2%)	7/40 (17.5%)	<0.05
2	15/38 (39.4%)	20/40 (50.0%)	>0.05
3	9/38 (23.7%)	9/40 (22.5%)	>0.05
4 (=Recurrence rate, number)	1/38 (2.6%)	4/40 (10.0%)	>0.05

a Mean ± SD (range).

### Final Appearance

Table 3 showed of three-quarters of those who received ethanol (73.6%) had final appearances that were classified either as grade 1 or 2 (i.e., appearance generally acceptable to both the patient and the physician), whereas around two-thirds of patients who underwent MMC treatment (67.5%) received a grade 1 or 2. The difference in the proportion of patients with cosmetically acceptable final appearance between the two groups was not statistically significant (*p*=0.56).

### Complications

As shown in Table 4, no complication was found in both groups treated with intraoperative ethanol, and 16.6% complication rates were found in MMC-treated group. Superficial punctate keratitis was the most common complication in each of the MMC-treated group. Other minor complications included granuloma and dellen formation noted during the first and second week after bare sclera excision with intraoperative MMC. Throughout postoperative follow-up time, there were no severe complications, such as scleral necrosis, in any patients of this study.

**Table 4:** Complications of Intraoperative ethanol and MMC for primary pterygium

	Group 1 (n=38)	Group 2 (n=36)

Superficial punctatate keratitis	0/38	5/36 (13.6%)
Conjunctival granuloma	0/38	1/36 (2.7%)
Formation of dellen	0/38	1/36 (2.7%)
Total	0/38 (0%)	6/36 (16.7%)

## DISCUSSION

In this study we have demonstrated the superiority of intraoperative ethanol compared to intraoperative MMC as an adjuvant therapy of pterygium excision in preventing recurrence. During a follow-up for a minimum of 12 months, we found there were no complications due to intraoperative 20% ethanol application. The recurrence rates of intraoperative MMC treatment (concentrations of 0.02-0.04% for 1-5 minutes) ranged from 3.3 to 42.9% in primary pterygium patients (49-52) and our data (0.025% for 1 minute) showed a recurrence rate of 11.1%.

In the past decade, mounting evidence has implied that many factors are involved in the process of pterygium formation including the UV-induced inflammatory cytokines (30), growth factors (20, 21, 23, 31-34) and proteolytic enzymes like matrix metalloproteinases and their inhibitors. (35) These factors could be secreted by cells of pterygium tissues, blood vessels, leukocytes and pterygium-adjacent corneal cells and closely related to the high recurrence rate of pterygium after surgical excision (30-35). These data suggest that therapy of alleviating these factors could possibly act as an adjuvant of surgical excision to prevent recurrence and we have demonstrated 20% ethanol is safe and effective. Though the difference was not significant, the complication rate is lower and corneal epithelial healing is faster in ethanol treatment group (*P*<0.05). Furthermore, an advantage of ethanol treatment is the underling stromal smoothness. As the electron microscope studies of LASEK (58), ethanol delamination of the pterygium/corneal epithelium results in a very smooth cleavage at the level of the pterygium/stromal attachments. (Figure 3) It leaves behind a very smooth surface, which is ideal for epithilaization.

Though the histological and pathological changes of pterygial tissues have been well studied, almost all of the documented specimens did not include the adjacent tissues. Seifert P et al. have demonstrated that the corneal tissues adjacent to the pterygial protuberance were histologically altered (53) It has been proposed that pterygia recur because of the incomplete removal of altered limbal cells that have invaded basal corneal, conjunctival, and circumferential limbal epithelia. (29) It follows that if all the histologically abnormal tissues (including ptergium and pterygium-adjacent tissues) are removed, the rate of recurrence will be reduced (53, 54). But in practice, for ophthalmologic surgeons it is hazardous to excise ocular tissues extensively for a benign lesion like pterygium because severe or vision-threatening complications such as corneal ectasia/perforation, post-operative astigmatism and corneal/scleral ulcers could result. By our data, the extended range, 1-1.5 mm central to the head of pterygium (Figure 3) and the surgical area, of 20% ethanol application could possibly be an effective method to alleviate the possible factors in pterygium-adjacent tissues that are highly related to recurrence.

Compatible with the reports on LASEK, no complications of low-dose ethanol treatment in our study were noted during the more than one year of follow-up. Though it was shown that after 20% ethanol treatment of the cornea for less than one minute, most epithelial cells were found alive (41), the occurrence of widespread partial or total damage of microvilli, focal breaks of intercellular junction, and cellular edema was observed by microscopy. (57) Others have observed no corneal complications in all the patients treated with ethanol of similar concentrations, 18 to 20%, for shorter durations, 30 to 45 seconds, in LASEK (53-55). On the basis of these findings, we have strong reasons to believe intraoperative low-dose ethanol application for one minute is safe, but more cases and a longer follow-up time are needed to achieve a solid conclusion. Therefore, we recommend a large-scale, randomized, controlled study of various dosage regimens to elucidate the optimal dosage and long-term safety profile of intra-operative ethanol.

On the contrary, toxicity of MMC is well-documented and can possibly manifest through vision-threatening complications such as scleral ulceration, corneal perforation, secondary glaucoma, and complicated cataract to minor side effects such as low-grade ocular pain, photophobia, superficial punctate keratitis and delayed conjunctival wound healing. (4, 10, 44, 51, 52, 55, 56) Intraoperative MMC delivery involves a direct sclera bed application of MMC that could possibly be a risk factor predisposing to the scleral ulcer. By our observations in the current study, we also found a significant longer time duration for corneal epithelial healing in MMC treatment group. During the 14-month mean follow-up of MMC group, we noted only early, minor side effects and no serious complications. This is probably due to the lower MMC concentration and shorter dose duration we adopted. (8)

In conclusion, the results of our study demonstrated that ethanol administered over a very short time interval intraoperatively (20% ethanol for 60 seconds) is a safe and effective way to reduce the pterygium recurrence rate after excision of pterygium. In the current study, the low pterygium recurrence rate, the high level of cosmetic satisfaction, the simplicity of the procedure, the minimal risk of minor side effects, and the lack of major complications after treatment lead us to recommend this regimen as an alternative for the treatment of pterygium, especially for those patients of high risk group for MMC treatment.
